# Psychosomatic complaints and sense of coherence among adolescents in a county in Sweden: a cross-sectional school survey

**DOI:** 10.1186/1751-0759-2-4

**Published:** 2008-02-08

**Authors:** Bo Simonsson, Kent W Nilsson, Jerzy Leppert, Vinod K Diwan

**Affiliations:** 1 Department of Community Medicine, County Council of Västmanland, Västerås, Sweden; 2 Centre for Clinical Research, Uppsala University, Central Hospital, Västerås, Sweden; 3 Department of International Health (IHCAR), Karolinska Institute, Stockholm, Sweden

## Abstract

**Background:**

Over the last five to ten years there has been an increase in psychosomatic complaints (PSC) in Swedish children. The objective of the study was to examine the relation between PSC and sense of coherence (SOC).

**Methods:**

A cross-sectional school survey in the county of Västmanland, Sweden. All 16- and 19-year old adolescents present at school on the day of the survey were asked to complete a questionnaire in their classrooms during a one-lesson hour session under the supervision of their teachers. Totally 3,998 students in both private and public schools, studying in ninth grade elementary school or third grade secondary school participated.

**Results:**

The results from our study show that there is a statistically significant relation between PSC and SOC among adolescents. It also shows that adolescents with a weak SOC score have more symptoms of PSC.

**Conclusion:**

Our study indicates that SOC can help the adolescents to choose a coping strategy that is appropriate for the situation and thereby may prevent them from developing PSC. However, additional studies are needed to confirm our findings.

## Introduction

The major causes of mortality and morbidity among adolescents have, during the last decades, shifted from infectious to behavioural aetiologies [1]. This shift is mostly due to risk taking behaviours among adolescents that lead to, among others, injuries and drug and alcohol misuse [2]. Mental health problems are also common among young people. The mental health problems can range from fully developed psychiatric diseases to learning disabilities, behavioural problems in school, speech- and language problems and psychosomatic complaints (PSC) [3]. To better understand the development of psychosomatic complaints among adolescents, it was decided to perform a population-based study, which is to our knowledge, the first to examine the relation between PSC and sense of coherence (SOC) among adolescents.

### Psychosomatic complaints

The symptoms of psychosomatic disorders usually begin during adolescence or early adulthood and are characterised by many vague physical complaints. Any part of the body may be affected, although the symptoms and their frequencies vary. Common symptoms among adolescents are headaches, nausea and vomiting, abdominal pain, diarrhoea or constipation, fatigue, fainting, dizziness, sleeping problems, and nervousness. The most frequently seen symptoms among adolescents should be differentiated from psychosomatic disorders seen among adults [4].

During the last decade, studies in several countries have shown that the proportion of adolescents with psychosomatic complaints (PSC) increased [5-9]. At present, it is estimated that between 5–10% of adolescents have so severe problems that they affect their daily lives. There are several social and structural factors that contribute to the perceived health among adolescents. One is the family structure. Among adolescents who live with single parents, PSC is twice as common, compared to adolescents living in nuclear families.

The Health Behaviour in School-Aged Children (HBSC) study, conducted by the World Health Organisation in 35 countries in 2002, indicates that adolescents from less privileged socio-economic groups report more physical and psychological symptoms than those from more privileged backgrounds [10]. In an interesting study, the physical symptoms and the psychological complaints in Japanese and Swedish schoolchildren was compared [11]. There was a significant difference between the two groups in terms of both physical symptoms and psychological complaints suggesting, that there might also be a cultural dimension to the development of PSC.

Current research suggests that psychological coping resources may serve as a buffer against the effects of negative stress on health. Antonovsky has developed a salutogenic model, the sense of coherence that indicates that successful coping with daily stressors is decisive for maintaining good health [12].

### Sense of coherence

According to Antonovsky, sense of coherence (SOC) is a way in which persons control their environment in order to make meaningful action [13]. SOC is a way of seeing the world, which facilitates successful coping with the innumerable, complex stressors that confront individuals in their daily lives. Furthermore, Antonovsky postulates that, "SOC is very explicitly not a substantive coping strategy, as mastery orientation or an internal locus of control". Individuals with a strong SOC perceive the world as predictable, manageable and meaningful and view environmental stressors as challenges worth facing. Such a person is very flexible, which enables him/her to find successful solutions to conflicts. A person with a strong SOC is less likely to perceive many stressful situations as threatening and anxiety provoking than one with a weak SOC. This way of conceptualising SOC can have certain implications when it comes to adolescents and psychosomatic symptoms [14].

This study is based on the results from an ongoing longitudinal survey known as "Survey of Adolescent Life in Vestmanland" (SALVe). The survey was started in 1995 to describe the health situation and secular trends in health, lifestyle and habits in the whole county of Västmanland in Sweden, a county situated in the middle of Sweden with 260,000 inhabitants. Included in the longitudinal survey are all students, in both public and private schools, attending 6^th ^and 9^th ^grade elementary school and 3^rd ^grade upper secondary school. Children in institutions or attending special schools for intellectually handicapped were excluded. The survey is unique in that it has surveyed a large number of students. So far 13,362 individual students have been included in the survey. Results from the longitudinal survey have been reported in several scientific articles [15-19]. Five cross-sectional studies have so far been conducted, in 1995, 1998, 2001, 2004 and 2006. These studies have shown that there is an increase in the proportion of adolescents who experience psychosomatic symptoms.

This study had the following objective: to test the hypotheses that: (i) individuals with weaker SOC will have more PSC; and, (ii) individuals with stronger SOC will have less PSC. Furthermore, our aim was also to test if our SOC-PSC-model controls for the psychosocial risk factors used in our study.

## Methods

### Study design and participants

For this study we used data from the cross-sectional study performed 1998. The questionnaires were distributed to all public and private schools in Västmanland. The students voluntarily and anonymously answered the questionnaire items during a 40–60 minute lesson-hour.

The inclusion criteria were that the participants should either attend 9^th ^grade in primary school or 3^rd ^grade secondary school in the County of Västmanland. The total eligible population was 5,125. Of these 4,305 completed the questionnaire. After adjusting for internal dropout, the total student population that participated in this study was 3,998 students, or 78.0% of the eligible students. Of the 820 students who did not complete the questionnaire, 463 students in 9th grade classes had planned practical working life orientation and were not at school the day the questionnaire was administered. Others, 98 students, were absent due to illness, 98 students, and the remaining 259 for other unknown reasons.

### Survey questionnaire

The questionnaire in the health study included several items from which the following were selected for this study: age, gender, social background factors, psychosomatic complaints and Antonovsky's short, 13-items version [Additional file 1].

The psychosocial background factors were ethnicity, residential type, family constellation and parental employment. The factors were coded as follows:

Ethnicity: (1) the adolescent was born in Sweden, (2) born abroad.

Residential type: (1) single family housing, (2) living in a block of flats.

Family constellation: (1) biological parents living together (2) separated parents.

Parental employment: (1) both parents working, (2) at least one non-working parent.

There were six physical symptoms measuring the psychosomatic complaints; i.e., headache, stomachache, feeling irritated, nervousness, sleeping problems and dizziness. These symptoms were all measured in four categories: often, sometimes, seldom and never. Each item was reversed for the index-calculation (high score = severe symptoms). A factor analysis was performed that gave a one-dimensional factor structure with an eigenvalue of 2.792. The six components explained 47% of the variance in the material. The factor loading for each component was for; stomach-ache .726, headache .695, dizziness .688, feeling irritated .684, nervousness .659 and for problems sleeping .638. Among boys, the corresponding eigenvalue was 2,758, variance explained 46% and component loading ranged between .650 – .725. Among girls, the eigenvalue was 2,501, variance explained 42% and component factor loading ranged between .603 – .686.

Thereafter a computation of an index of psychosomatic symptoms was done to find adolescents with several PSC. Also, the index was dichotomised into few symptoms and severe symptoms (> 1 S.D. from mean). Each item was also dichotomised to often/sometimes and seldom/never.

When answering the items in Antonovsky's short, 13-items version, each individual receives a score ranging from 13 to 91. Four equally large groups were created: strong, fairly strong, fairly weak and weak SOC: to incorporate a SOC in the hypothesised model.

The short version of the SOC scale has been shown in a systematic review of 127 studies to have a Cronbach's α with a range from 0.70–0.92 and a one-year test-retest correlation of 0.69–0.78 [20]. In the present study the Cronbach's α for all 13 items were 0.860.

This study has followed the recommendations and principles of the Swedish Council for Working Life and Social Research. In summary, the recommendations include that a person, who participates in research as a subject should be protected against the risk of physical injury, mental injury or the violation of their integrity. To the extent that the research can involve risks for the subjects of the research, there should be an investigation that includes, among other things, a weighing-up of the risks involved against the knowledge gained. High standards should be insisted upon with respect to the quality of the research and to ensure that the subjects involved have understood and accepted the conditions that apply to their participation.

### Statistical analysis

There were five statistical procedures performed using SPSS (Statistical Package for Social Sciences) version 15.0 for Windows:

A χ^2 ^test to determine if there was an association between the psychosocial background factors and psychosomatic complains.

Bivariate correlation procedure computing Pearson's correlation coefficient or Pearson's r to determine if there was a covariance between the psychosomatic symptoms.

An one-way ANOVA (post hoc. Tukey's test) to test if the means in the psychosomatic index varied with the quartiles in SOC.

Factor analysis to analyse the relation between the psychosomatic variables to identify groups with similar symptoms.

Binary logistic regression to control for the influence of various social conditions. The logistic regression resulted in an odds ratio. The R^2^coefficient, a measure of strength of the association in the models used in this study was Nagelkerke R^2^.

## Results

The distribution of psychosocial background factors is shown in Table 1. Approximately the same proportion of adolescents had families in which both parents and the child came from abroad (7,0%) as were born in Sweden with foreign parents (5,9%). Roughly one out of four (27%) of the adolescents lived in block of flats and three out of ten (33%) came from families with separated parents. Parental unemployment was reported by 14–15% of the adolescents.

**Table 1:** Social background factors among 2023 boys and 1975 girls 16 and 19 year-old in Västmanland County, Sweden

Background factors	Boys N = 2023	(%)	Girls N = 1975	(%)
Ethnicity				
Parents and child from Sweden	1664	82.3	1645	83.3
Child born in Sweden, parents from abroad	117	5.8	120	6.1
Both child and parents from abroad	151	7.5	127	6.4
				
Residential type				
Private residence	1458	72.1	1432	72.5
Rented flat or apartment	557	27.5	531	26.9
				
Family constellation				
Nuclear family	1335	66.0	1333	67.5
Separated family	677	33.5	638	32.3
				
Parental employment				
Both parents working	1629	80.5	1587	80.4
At least one non-working parent	297	14.7	287	14.5

Note: Numbers may vary slightly due to missing data for specific variables

The distribution, by gender and age, of the reported symptoms is described in Table 2. There was a gender difference in the symptoms reported, with the boys reporting fewer and less frequent symptoms than the girls. The most common symptoms among the girls were headache and feeling irritated, while the boys reported feeling irritated and having sleeping problems.

**Table 2:** Number and proportion of reported psychosomatic complaints among 16 and 19 year-old boys and girls, Västmanland County, Sweden

	Boys 16 years				N = 1078				Boys 19 years				N = 964			
	Often		Sometimes		Seldom		Never		Often		Sometimes		Seldom		Never	
	n	%	n	%	n	%	n	%	n	%	n	%	n	%	n	%
Headache	76	7	312	29	462	43	228	21	76	8	253	27	405	42	219	23
Stomachache	34	3	245	23	502	47	297	28	57	6	179	19	415	43	302	32
Feeling irritated	175	16	439	41	333	31	131	12	141	15	411	43	305	32	96	10
Nervousness	59	5	311	29	468	43	240	22	56	6	279	30	431	45	187	20
Problem sleeping	117	11	219	20	339	31	403	37	115	12	208	22	338	35	292	31
Dizziness	54	5	133	12	354	33	532	50	24	3	104	11	292	31	530	56
																
	Girls 16 years				N = 1029				Girls 19 years				N = 953			
	Often		Sometimes		Seldom		Never		Often		Sometimes		Seldom		Never	
	n	%	n	%	n	%	n	%	n	%	n	%	n	%	n	%

Headache	194	19	459	45	297	29	79	8	250	26	399	41	243	25	72	7
Stomachache	127	12	441	43	368	36	93	9	174	18	355	37	353	37	82	8
Feeling irritated	285	28	491	48	213	21	40	4	280	29	499	52	164	17	21	2
Nervousness	129	12	382	37	407	40	111	11	164	17	393	41	330	34	77	8
Problem sleeping	139	13	241	23	352	34	297	29	176	18	281	29	301	31	206	21
Dizziness	86	8	173	17	345	34	406	40	72	8	200	21	310	32	379	39

The distribution of psychosomatic symptoms related to psychosocial background factors varied with gender, as can be seen in Table 3. According to the severe PSC symptom dichotomization (>1 SD) there were significantly more girls affected by PSC, (χ^2 ^= 179.322, df = 1, p < 0.001) Among the boys, 9% have regular PSC as compared to 25% among the girls. In our study we found that severe PSC (> 1 S.D. from mean) among both boys and girls was significantly related to all the psychosocial background factors, i.e. ethnicity, residential type, family constellation and parental employment.

**Table 3:** Social background factors associated with severe psychosomatic symptoms among 16 and 19-year old boys and girls Västmanland county, Sweden

Background factor	Severe psychosomatic symptoms >1 SD from mean			
	
	Boys N = 2023		Girls N = 1975	
	
	n	% (C.I)	n	% (C.I)
**Total**	190	9.4 (8.1–10.7)	502	25.4 (23.4–27.4)
				
**Ethnicity**				
Parents and child from Sweden	146	8.8 (7.4–10.2)	407	24.7 (22.6–26.8)
Child born in Sweden parents from abroad	9	7.7 (2.9–12.5)	31	25.8 (18.0–33.6)
Both child and parents from abroad	24	15.9 (10.0–21.8)	43	33.9 (25.7–42.1)
				
**Residential type**				
"Own house"	122	8.4 (7.0–9.8)	328	22.9 (20.7–25.1)
Flat or apartment	66	11.8 (9.1–14.5)	173	32.6 (28.6–36.8)
				
**Family constellation**				
"Nuclear family"	112	8.4 (6.9–9.9)	320	24.0 (21.7–26.3)
Separated family	77	11.4 (9.0–13.8)	180	28.2 (24.7–31.7)
				
**Parental employment**				
Working parents	142	8.7 (7.3–10.1)	380	23.9 (21.8–26.0)
At least one non-working parent	39	13.1 (9.3–16.9)	95	33.1 (30.8–35.4)

Note: Numbers may vary slightly due to non-response for specific variables

Besides the difference in the reported prevalence of symptoms, there was also a gender difference in the SOC scores. The girls scored lower than the boys in both age groups. The sixteen-year-old boys had a mean SOC score of 61.10 (S.D. = 12.14) while the nineteen-year-old boys had a mean score of 62.5 (S.D. = 12.33). The girls had a mean score of 59.0 (S.D. = 13.41) for the sixteen year olds and the nineteen-year-old girls had a mean score of 58.5 (S.D. = 12.01).

A bivariate correlation procedure computing Pearson's correlation coefficient or Pearson's r to determine the covariance between the psychosomatic symptoms was done. There was a weak association, between the psychosomatic symptoms, with values ranging between .489 and .281. The symptoms grouped themselves as follows: headache to stomach ache, feeling irritated to nervousness and problem sleeping to dizziness.

Table 4 illustrates that the stronger the SOC the lesser proportion of adolescents that can be classified as having severe PSC and vice versa (p < 0.001 among both sexes). Almost one out of ten boys and just over one out of four girls reported having severe PSC. In addition, in each SOC-subgroup girls had more than double proportions of severe PSC than boys. In the group of adolescent with the weakest SOC, more than 21% of the boys reported to have severe PSC in comparison to the more than 51% of the girls.

**Table 4:** Sense of Coherence associated with severe psychosomatic symptoms among 16 and 19-year-old boys and girls in Västmanland County, Sweden.

SOC	Severe psychosomatic symptoms >1 SD from mean				Sense of coherence score limits classified by quartile	
	
	Boys		Girls		Boys	Girls
	
	n	%	n	%		
Total	190	9.4	502	25.4		
Strong SOC	10	1.8	45	9.2	73–91	70–91
Fairly strong SOC	16	3.2	72	14.1	63–72	60–69
Fairly weak SOC	53	11.3	121	26.1	54–62	51–59
Weak SOC	111	21.7	264	51.6	13–53	16–50
		*		*		

* = p < .001

The one-way ANOVA (post hoc Tukey's test) showed that there was a significant association between the SOC quartiles and the psychosomatic index as shown in Table 5.

**Table 5:** Relation between mean SOC and PSC index. One way ANOVA (post hoc. Tukey's test)

	Boys		Girls		Total
	
	16 years of age	19 years of age	16 years of age	19 years of age	
Strong SOC	10,9769*	11,0405*	12,6360*	13,6467*	11,8765*
Fairly strong SOC	12,561*	12,2895*	14,4248*	14,5226*	13,3998*
Fairly weak SOC	13,77657*	13,3457*	15,5086*	15,7556*	14,5682*
Weak SOC	14,91213*	14,9742*	16,9412*	17,9848*	16,4466*

* = p < .01

Table 6 shows the logistic analyses that were performed using the two models. In the first model, model A, SOC was tested in relation to PSC, and in model B, adjustment for psychosocial background factors was made. In the logistic model A, we found that the category of adolescents that reported many and severe psychosomatic complains was significantly related to SOC-quartiles. The same pattern was found when the model was adjusted for psychosocial background factors; model B. All psychosocial background factors had a non-significant relation too psychosomatic complaints when adjusted for SOC, except among girls from blocks of flats who had an increased risk for psychosomatic complaints (p = 0.046 OR 1.3) (not shown in figure). As shown in the table, adolescent boys with weak SOC had an increased risk of 14.7 times (unadjusted) to 16.4 times (adjusted) for experiences of severe PSC in comparison with boys with strong SOC (the reference category). The corresponding elevated risk for girls with weak SOC was 9.9 (adjusted) and 10.5 (unadjusted). Our SOC-models explained 16–20% of severe psychosomatic complaints present among the 16- and 19-year-old adolescents.

**Table 6:** Logistic regression analysis of two models of Sense of Coherence and psychosocial complains in boys and girls in Västmanland county, Sweden.

	Many/severe psychosocial complains Boys			Many/severe psychosocial complains Girls		
Sense of Coherence	n	Odds ratio Model A (C.I.)	Odds ratio Model B (C.I.)	n	Odds ratio Model A (C.I.)	Odds ratio Model B (C.I.)

Strong	541	1.0	1.0	490	1.0	1.0
Medium strong	502	1.7 (0.8–3.9)	2.1 (0.8–4.9)	509	1.6 (1.1–2.4)	1.7 (1.1–2.6)
Medium weak	469	6.8 (3.4–13.5)	7.3 (3.4–15.7)	464	3.5 (2.4–5.1)	3.5 (2.4–5.2)
Weak	511	14.7 (7.6–28.5)	16.4 (7.9–34.3)	512	10.5 (7.4–15.0)	9.9 (6.8–14.3)
		R^2 ^.157	R^2 ^.167		R^2 ^.196	R^2 ^.193

R^2 ^Nagelkerke

Models

A) No adjustment

B) Adjusted for ethnicity, residential type, family constellation, parental employment

## Discussion

The results from our study showed that there was a statistically significant association between PSC and SOC, and also that lower SOC was associated with a higher prevalence of PSC. Adolescent boys with a weak SOC had an increased risk for severe psychosomatic symptoms, 15 times higher than boys with a high SOC. The corresponding risk-elevation among girls was 10 times. Conversely, higher SOC was associated with a lower prevalence of PSC. The findings in our study suggest that a strong SOC can help adolescents choose a coping strategy that is appropriate for the situation and thereby may prevent them from developing PSC, which is in line with other authors findings [21,22].

Our results also indicate that the psychosocial background factors used in this study do not in any meaningful way explain the variation in psychosomatic complains, when controlling for SOC. Instead, the reverse pattern was found in which the SOC variable out-ruled the traditional psychosocial risk factors in our model. Consequently, this does not exclude the possibility that there are other psychosocial factors that could influence or explain the variation in PSC.

In an earlier study it was found that 25% of the children in the Nordic countries had at least one PSC occurring every week or every other week, with a higher prevalence among girls [23]. The most common complaints were headache and stomachache. It also showed that there are several factors related to PSC. These factors can be found at the child, family and socio-economic level [13]. In our study, we also found the same pattern with headache and stomachache as the most common complaints, of gender difference, with more PSC among girls. The proportion of adolescents that reported PSC was similar to the proportion found in the aforementioned study. In an unpublished study, we found that the proportion of girls with PSC increased from 14 to 21% during a 10-year period, while there was no increase in PSC among the boys.

Total SOC scores are normally lower for women than for men. The reason for this is not clear. However it has been suggested that the SOC scale could be based on a way of reasoning that is more relevant for women than men, as men and women have different coping strategies. Men tend to be more aggressive and presumptuous, while women are more reflective and emotional [24].

McSherry and Holm showed in their study that not only are low SOC individuals more psychologically distressed prior to a stressful situation, but they also maintained these higher levels of distress after stressful experience as well [25,26]. Although the levels of distress showed significant pre and post increases for all three SOC groups, the low SOC group began and ended the experiment as the most distressed. This could be a part of the explanation why individuals with low SOC develop more psychosomatic symptoms, as the results from our study have shown.

Geyer discussed in his paper the statistical relationship between SOC and symptoms/disease [27]. He concluded that due to the simultaneous assessment of variables, it is open to debate whether a low SOC has some effect on the probability of falling ill or whether it is the other way around.

Our results indicate that SOC has an effect on the development of psychosomatic symptoms, as almost no psychosocial background factors explained any difference, when controlling for SOC. Instead, all psychosocial factors had significant relation to PSC if not controlled for SOC.

SOC could be interpreted as an autonomous resource contributing to a favourable development of subjective state of health. SOC data should; however, be regarded as complementary to and not a substitute for information already known to be associated with increased risk of future ill health [27]. Other authors report that SOC was not found to be a buffering variable [28].

All these measures are self-reported and therefore subject to reporting bias. Self-reported data are also considered to have questionable validity in many cases, especially survey questions about socially unacceptable behaviour. Questions about somatic and psychological symptoms are also difficult to validate in a cross-sectional study with a potential for under- or over-reporting that could lead to an inflation of the estimates of PSC in our study. Complementary interviews would have been preferred, but this was not feasible due to the large sample size and the confidential nature of the survey. There were no missing data analyses performed.

The strengths of this study are the large sample, the high response rate and the use of commonly used and tested measures. As the data was sampled during a lesson-hour under the surveillance of a teacher, the situation was controlled. However, with an external non-response rate of 16% (820 students) and an internal non-response rate that varied between 1 to 3% the students not attending the school during the period the survey was conducted may introduce a response bias. Non-respondents may also be more likely to suffer from PSC, hence leading to less statistical power in detecting this potential response bias.

The data from the adolescents in this study represent only one county in Sweden and this may limit our ability to make generalisations. The county does not, however, differ from other counties with regard to socio-economic or demographic factors.

Our results only represent relations among variables and not causations. The relations presented are associative, given that conclusions regarding the directions of cause and effect must be considered tentative. It is possible that a person who developed severe PSC has weakened ability to develop a strong SOC or coping ability, or that there are other, yet unknown factors that might be related to both SOC and PSC. These factors could be a genetic predisposition or environmental or cultural influences, as well as other psychosocial factors than those used in our study. As this study is one of the first studies to examine the association between SOC and PSC in an adolescent population, both a replications of this study and these analyses in different settings would be needed as is longitudinal study to follow the secular trend of PSC in adolescents.

In summary our study shows that adolescents with weak SOC report more psychosomatic symptoms than adolescents with a strong SOC. We also found that SOC ruled out traditional psychosocial risk factors, indicating that SOC is an intervening variable that mediates psychosocial risks in relation to PSC.

## Abbreviations

SOC, sense of coherence; PSC, psychosomatic complaints; S.D., standard deviation.

## Competing interests

The author(s) declare that they have no competing interests.

## Authors' contributions

BS drafted the manuscript and performed the analyses of the study. KWN was responsible for designing the study and statistical analyses. JL supervised the design of the study and VKD supervised the writing of the manuscript and the analyses of the results. All authors have read and approved the final manuscript.

## Supplementary Material

Additional file 1 Antonovsky's short 13-item version of the Sense of coherence scale. The concise version of Antonovsky's short 13-item questionnaire. Click here for file
