# Erratum to: Psychosomatic complaints and sense of coherence among adolescents in a county in Sweden: a cross-sectional school survey

**DOI:** 10.1186/s13030-015-0050-4

**Published:** 2015-11-06

**Authors:** Bo Simonsson, Kent W. Nilsson, Jerzy Leppert, Vinod K. Diwan

**Affiliations:** Department of Community Medicine, County Council of Västmanland, Västerås, Sweden; Centre for Clinical Research, Uppsala University, Central Hospital, Västerås, Sweden; Department of International Health (IHCAR), Karolinska Institute, Stockholm, Sweden

The Additional file 1 in this article [1] has been removed at the request of the copyright holder. Information about Antonovsky’s short 13-item version of the Sense of Coherence scale is available at http://www.salutogenesis.hv.se/eng/SOC_questionnaire.19.html.
