# Supplementary material for: Psychosomatic complaints and sense of coherence among adolescents in a county in Sweden: a cross-sectional school survey
Source: Biopsychosoc Med. 2008 Feb 8;2:4. doi: 10.1186/1751-0759-2-4 (PMC2265297; doi:10.1186/1751-0759-2-4)
Supplement: Additional file 1 — Antonovsky's short 13-item version of the Sense of coherence scale. The concise version of Antonovsky's short 13-item questionnaire. [file 1751-0759-2-4-S1.doc]

**The 13-item Sense of Coherence Questionnaire**

Here is a series of questions relating to various aspects of your lives. Each question has seven possible answers. Please mark the number, which expresses your answer, with number 1 and 7 being the extreme answers. If the words under 1 are right for you, circle 1: if the words under 7 are right for you, circle 7. If you feel differently, circle the number which best expresses your feeling. Please give only one answer to each question.

1. Do you have feeling that you don’t really care about what goes on around you?

| 1 | 2 | 3 | 4 | 5 | 6 | 7 |
| --- | --- | --- | --- | --- | --- | --- |

very seldom very often or never

2. Has it happened in the past that you were surprised by the behaviour of people whom you thought you knew well?

| 1 | 2 | 3 | 4 | 5 | 6 | 7 |
| --- | --- | --- | --- | --- | --- | --- |

never happened always happened

3. Has it happened that people whom you counted on disappointed you?

| 1 | 2 | 3 | 4 | 5 | 6 | 7 |
| --- | --- | --- | --- | --- | --- | --- |

never happened always happened

4. Until now your life has had:

| 1 | 2 | 3 | 4 | 5 | 6 | 7 |
| --- | --- | --- | --- | --- | --- | --- |

no clear goals very clear

or purpose at all goals and purpose

5. Do you have the feeling that you’re being treated unfairly?

| 1 | 2 | 3 | 4 | 5 | 6 | 7 |
| --- | --- | --- | --- | --- | --- | --- |

very often very seldom or never

6. Do you have the feeling that you are in an unfamiliar situation and don’t know what to do?

| 1 | 2 | 3 | 4 | 5 | 6 | 7 |
| --- | --- | --- | --- | --- | --- | --- |

very often very seldom or never

7. Doing the thing you do every day is:

| 1 | 2 | 3 | 4 | 5 | 6 | 7 |
| --- | --- | --- | --- | --- | --- | --- |

a source of deep a source of

pleasure and pain and

satisfaction boredom

8. Do you have very mixed-up feelings and ideas?

| 1 | 2 | 3 | 4 | 5 | 6 | 7 |
| --- | --- | --- | --- | --- | --- | --- |

very often very seldom or never

9. Does it happen that you have feelings inside you would rather not feel?

| 1 | 2 | 3 | 4 | 5 | 6 | 7 |
| --- | --- | --- | --- | --- | --- | --- |

very often very seldom or never

10. Many people – even those with a strong character – sometimes feel like sad sacks (losers) in

certain situations. How often have you felt this way in the past?

| 1 | 2 | 3 | 4 | 5 | 6 | 7 |
| --- | --- | --- | --- | --- | --- | --- |

never very often

11. When something happened, have you generally found that:

| 1 | 2 | 3 | 4 | 5 | 6 | 7 |
| --- | --- | --- | --- | --- | --- | --- |

you overesti- you saw

mated or under- things in the

estimated its right

importance proportion

12. How often do you have the feeling that there’s little meaning in the things you do in your

daily life?

| 1 | 2 | 3 | 4 | 5 | 6 | 7 |
| --- | --- | --- | --- | --- | --- | --- |

very often very seldom or never

13. How often do you have feelings that you’re not sure you can keep under control?

| 1 | 2 | 3 | 4 | 5 | 6 | 7 |
| --- | --- | --- | --- | --- | --- | --- |

very often very seldom
